# Current trends in the treatment of pneumonia due to multidrug-resistant Gram-negative bacteria

**DOI:** 10.12688/f1000research.16517.2

**Published:** 2019-02-06

**Authors:** Richard R. Watkins, David Van Duin

**Affiliations:** 1 Division of Infectious Diseases, Cleveland Clinic Akron General, Akron, OH, 44302, USA; 2 Department of Medicine, Northeast Ohio Medical University, Rootstown, OH, 44272, USA; 3 Department of Medicine, University of North Carolina, Chapel Hill, NC, 27514, USA

**Keywords:** Enterobacteriaceae, Pseudomonas aeruginosa, Acinetobacter baumannii, antibiotics

## Abstract

Pneumonia is one of the most common infections worldwide. Morbidity, mortality, and healthcare costs increase substantially when pneumonia is caused by multidrug-resistant Gram-negative bacteria (MDR-GNB). The ongoing spread of antimicrobial resistance has made treating MDR-GNB pneumonia increasingly difficult. Fortunately, there have been some recent additions to our antibiotic armamentarium in the US and Europe for MDR-GNB, along with several agents that are in advanced stages of development. In this article, we review the risk factors for and current management of MDR-GNB pneumonia as well as novel agents with activity against these important and challenging pathogens.

## Introduction

Infections due to multidrug-resistant Gram-negative bacteria (MDR-GNB) pose a serious and increasing threat to human health. This is particularly true in the pathogenesis of pneumonia, where escalating rates of antimicrobial resistance (AMR) have been associated with excessive morbidity, mortality, and healthcare costs ^1^. It is well recognized that timely and effective therapy is vital for improving outcomes, especially for pneumonia that is hospital acquired ^2^. The choice of initial antibiotic therapy for pneumonia is based on several factors, including recommendations from guidelines, national and local antimicrobial susceptibility data, and patient characteristics such as allergies and renal function. For many years, the backbone of treatment for pneumonia has been the β-lactam class of antibiotics, including the third- and fourth-generation cephalosporins and β-lactam/β-lactamase inhibitor combinations like piperacillin/tazobactam ^3^. Unfortunately, the ongoing spread of extended-spectrum β-lactamases (ESBLs) and carbapenemases such as *Klebsiella pneumonia* carbapenemase (KPC) has begun to limit the clinical effectiveness of β-lactam agents over the last decade ^4, 5^.

The diagnosis of pneumonia can be challenging, especially in cases of hospital-acquired pneumonia (HAP) and ventilator-associated pneumonia (VAP). Indeed, pulmonary infiltrates on imaging in critically ill patients are common and can be due to non-infectious etiologies, including atelectasis, acute respiratory distress syndrome (ARDS), congestive heart failure (CHF), pulmonary hemorrhages, and pulmonary infarction. Moreover, upper airways and endotracheal tubes of hospitalized patients are often colonized by MDR-GNB and their presence does not necessarily mean that they are the cause of the pulmonary abnormalities seen on imaging studies. A careful clinical assessment is therefore imperative when evaluating for pneumonia, especially in patients who have had a prolonged hospitalization. The current HAP/VAP guidelines from the Infectious Diseases Society of America are an excellent reference for help with diagnosing these cases ^2^.

The initial approach to pneumonia is most often empirical because results of antimicrobial susceptibility testing typically take 48 to 72 hours. Rapid diagnostic tests (RDTs), including molecular methods that identify specific resistance genes or automated microscopy that can quickly determine antibiotic susceptibility, have great potential for guiding empiric antibiotic therapy. But current RDTs have limitations and most have not been validated for respiratory secretions ^6^. Deciding on an appropriate empirical regimen can be difficult because clinicians must consider the benefits of starting therapy early versus the harms of unnecessary coverage. Indeed, inappropriate antimicrobial treatment or delays in starting appropriate treatment in VAP are associated with increased morbidity and mortality ^7^. Once susceptibility testing results are available, empiric antibiotic therapy should be de-escalated. Most cases of MDR-GNB pneumonia can be successfully treated with 7 days of therapy ^2^.

Several risk factors for MDR-GNB pneumonia have been identified. These include prior infection or colonization with MDR-GNB, antibiotic therapy in the past 90 days, poor functional status performance, hospitalization for more than 2 days in the past 90 days, occurrence 5 or more days after admission to an acute hospital, receiving hemodialysis, and immunosuppression ^8, 9^. Moreover, prior receipt of carbapenems, broad-spectrum cephalosporins, and fluoroquinolones has been associated specifically with MDR *Pseudomonas aeruginosa* ^10^.

Recently, the high mortality and mortality associated with MDR-GNB pneumonia along with limited treatment options have led to a resurgence in the use of the nephrotoxic drug colistin ^11^. Fortunately, several new antibiotic agents with activity against MDR-GNB, including plazomicin, ceftazidime/avibactam, and meropenem/vaborbactam, have become available. This review discusses new antibiotic options for MDR-GNB and those in late stages of clinical development and provides guidance for their use in treating MDR-GNB pneumonia.

## Multidrug-resistant Enterobacteriaceae

Similar to other MDR-GNB, MDR Enterobacteriaceae are usually encountered as the cause of healthcare-associated pneumonia and are less commonly seen in community-acquired pneumonia (CAP). In a recent large intensive care unit (ICU) study of 75 US centers, the most common Enterobacteriaceae isolated from patients with pneumonia were *Klebsiella pneumoniae*, *Enterobacter* spp., and *Escherichia coli*, which accounted for 12%, 8%, and 7% of all bacterial isolates included in the study, respectively ^12^. Important MDR Enterobacteriaceae that cause pneumonia include those bacteria that produce AmpC enzymes, ESBL, or carbapenemases or a combination of these. AmpC and ESBL producers are usually resistant to most, if not all, cephalosporins. ESBL but not AmpC producers are variably inhibited by β-lactamase inhibitors. Also, AmpC enzymes are frequently found in *Enterobacter* spp. and may be induced by antibiotic treatment, leading to treatment-emergent resistance ^13^. As AmpC enzymes do not effectively hydrolyze cefepime, AmpC-producing Enterobacteriaceae often retain *in vitro* susceptibility to cefepime ^13^. The management of pneumonia caused by carbapenem-resistant Enterobacteriaceae (CRE) is the most challenging. In a longitudinal cohort study of patients with CRE, pneumonia and bloodstream infections (BSIs) were found to be associated with the highest mortality rates ^14^. When compared with comparable patients colonized with CRE, CRE pneumonia had an excess hospital mortality of 27% and adjusted hazard ratio of 3.44 (95% confidence interval [CI] 1.80–6.48, *p*<0.001) for time to death ^14^. Risk factors for MDR Enterobacteriaceae as a cause of pneumonia overlap with those of other MDR organisms and include prior exposure to antibiotics, healthcare exposure, and use of medical devices such as urinary catheters ^15, 16^.

An excellent comprehensive review on the therapy of MDR Enterobacteriaceae was recently published by Rodríguez-Baño *et al*. and the reader is referred to this review for additional background ^17^. It is important to note that the MERINO trial (Meropenem versus piperacillin-tazobactam for definitive treatment of bloodstream infections due to ceftriaxone non-susceptible *Escherichia coli* and *Klebsiella* spp.) had not yet been published at the time of that review. In the MERINO trial, patients with BSI caused by ceftriaxone-resistant Enterobacteriaceae were randomly assigned to receive either piperacillin/tazobactam or meropenem in an open-label non-inferiority design ^18^. The mechanism of resistance in these isolates was an ESBL in about 85% and AmpC in about 10%. In contrast to some observational studies, the mortality in piperacillin/tazobactam-treated patients was significantly higher as compared with those treated with meropenem (12% versus 4%) ^18, 19^. A total of 12 out of 379 patients had pneumonia as a source of BSI in this study. Similarly, in a randomized controlled trial comparing cefepime versus imipenem in the treatment of pneumonia, a decreased efficacy of cefepime was noted in patients infected with ESBL-producing Enterobacteriaceae ^20^. Based on these data, carbapenems should be considered first-line treatment for pneumonia caused by ESBL-producing Enterobacteriaceae. In an observational study of AmpC-producing Enterobacteriaceae BSI, patients who received definitive therapy with piperacillin/tazobactam were compared with a group of patients who received either cefepime or piperacillin/tazobactam. Only 14% and 20% of patients had pneumonia as the source of BSI ^21^. Although no statistically significant differences were found, it was notable that the 30-day mortality in the piperacillin/tazobactam-treated patients was 15% versus 7% in the meropenem- or cefepime-treated patients ^21^. Based on these clinical data and the known hydrolysis of piperacillin by AmpC, piperacillin/tazobactam cannot be considered first-line treatment for AmpC-producing Enterobacteriaceae. As mechanisms of resistance are usually not available to treating clinicians, practically speaking, piperacillin/tazobactam should not be used for pneumonia caused by *Enterobacter* spp., *Serratia* spp., or *Citrobacter* spp.

Whether cefepime can be successfully used in the treatment of pneumonia caused by AmpC-producing Enterobacteriaceae remains unclear. In a propensity-adjusted analysis of observational data on patients with infections caused by AmpC-producing Enterobacteriaceae, treatment with cefepime (n = 32) was compared with meropenem (n = 32) ^22^. Pneumonia was present in 53% and 41% of cefepime- and meropenem-treated patients, respectively. Overall, no difference in 30-day mortality was seen (odds ratio [OR] 0.63, 95% CI 0.23–211), but it should be noted that this OR had a wide CI that included both a fourfold decreased odds of 30-day mortality in cefepime-treated patients and a twofold increased odds ^22^. Given the absence of activity of AmpC against cefepime and these limited clinical results, cefepime is a reasonable carbapenem-sparing option for pneumonia caused by AmpC-positive Enterobacteriaceae.

Carbapenem resistance in Enterobacteriaceae can be mediated through carbapenemases, which are usually carried on mobile genetic elements such as plasmids (carbapenemase-producing Enterobacteriaceae, or CPE), or through a variety of other mechanisms such as porin mutations (non-carbapenemase-producing CRE). Important classes of carbapenemases include KPC, New Delhi metallo-β-lactamases (NDMs), and OXA-48-like carbapenemases ^23^. Prior to the availability of newer antibiotics, treatment of invasive CRE infections included the use of polymyxins, tigecycline, and aminoglycosides, often given in combination regimens ^24^. More recently, meropenem/vaborbactam, ceftazidime/avibactam, eravacycline, and plazomicin have become available ^25– 27^. These agents have specific *in vitro* anti-CRE activity. Plazomicin has broad activity regardless of carbapenemase but is inactivated by 16S rRNA ribosomal methyltransferases that are present in some NDM-producing CPE ^28^. Meropenem/vaborbactam is active against KPC-producing CPE, and avibactam inhibits KPC and OXA-48-like carbapenemases ^25, 29^. Eravacycline is a fluorocycline antibiotic similar in structure to tigecycline with activity against CRE ^30^. In addition, there are several other anti-CRE agents in the pipeline, including cefiderocol, imipenem/relebactam, and meropenem/nacubactam.

With the availability of these new agents, there are some (but no definitive) clinical data available on the best treatment of CRE infections, including CRE pneumonia. In an observational study, patients with CRE infections—caused primarily by KPC producers—who were started on colistin (n = 99) were compared with patients who were started on ceftazidime/avibactam (n = 38) ^31^. In an inverse probability of treatment-weighted analysis, the 30-day mortality in patients on colistin was 32% versus 9% (absolute difference 23%, 95% CI 9%–35%; *p* = 0.001) in those on ceftazidime/avibactam. Pneumonia was present in 24% and 32% of patients on colistin and ceftazidime/avibactam, respectively ^31^. In a randomized controlled trial, plazomicin was compared with colistin—both given in combination with either tigecycline or meropenem—in CRE BSI (n = 29) or pneumonia (n = 8) ^32^. Plazomicin versus colistin therapy was associated with 2/17 (12%) versus 8/20 (40%) all-cause mortality at day 28 ^32^. Similarly, meropenem/vaborbactam (n = 28) was compared with best available therapy (n = 15) for CRE infections, 5 of which were pneumonia ^26^. Rates of clinical cure at test of cure visit were 57% and 27% in the meropenem-vaborbactam and best available therapy arms, respectively ^26^. Based on these data, it is clear that polymyxin-based therapy is inferior to treatment with one of these novel agents. Although only limited numbers of patients with CRE pneumonia were studied, the use of either meropenem-vaborbactam or ceftazidime-avibactam in CRE pneumonia is a reasonable approach while awaiting more data. Ceftazidime/avibactam was shown to be non-inferior to meropenem in a recent large randomized controlled trial of non-CRE pneumonia ^33^. Meropenem itself has been used as a comparator agent in many pneumonia studies, and vaborbactam achieves high epithelial lining fluid concentrations ^34^. In contrast, in the absence of confirmatory data, plazomicin should not be considered a first-line choice for monotherapy of CRE pneumonia.

## *Pseudomonas aeruginosa* 

The acquisition of MDR *P. aeruginosa*, a significant and increasing cause of HAP/VAP in North America and Europe, is related to both patient factors (for example, older age, previous colonization, recent broad-spectrum antibiotic use, malignancy, and presence of shock) and nosocomial factors (for example, admission to a ward with a high incidence of MDR strains) ^35^. Indeed, recent receipt of an anti **-**pseudomonal antibiotic, especially quinolones and carbapenems, appears to be an important driver of MDR *P. aeruginosa* acquisition ^10^. Compared with less-resistant strains, pneumonia due to MDR *P. aeruginosa* is associated with longer stays in the ICU, prolonged mechanical ventilation, and greater mortality ^36^. Thus, improving the management of MDR *P. aeruginosa* pneumonia must be a priority in order to improve outcomes from both clinical and financial standpoints.

At present, there is no high-grade evidence to guide management decisions for MDR *P. aeruginosa* pneumonia. Current guidelines recommend combination empiric therapy when AMR is a concern and suggest that aminoglycosides and colistin be avoided if alternative agents are available (low-quality evidence) ^2^. An anti-pseudomonal cephalosporin, carbapenem, fluoroquinolone, or β-lactam/β-lactamase inhibitor is a potential option for initial therapy. Once susceptibility results are available, combination therapy can be de-escalated to monotherapy in most cases. However, combination therapy should be continued for patients in septic shock or at a high risk for death ^2^. Patients with VAP due to MDR *P. aeruginosa* that is susceptible to only aminoglycosides or polymyxins should receive both inhaled and systemic antibiotics rather than systemic antibiotics alone ^2^. In addition to adequate antibiotic coverage, other factors such as adequate drug dosing, appropriate intervals of drug administration, and duration of therapy (usually 7 days) are important for achieving optimal clinical outcomes. For example, a single-center retrospective study found that mortality was significantly lower in patients with *P. aeruginosa* pneumonia who received extended-infusion cefepime versus standard dosing (20% versus 3%, respectively; *p* = 0.03), along with significantly lower length of ICU stay (18.5 versus 8 days, respectively; *p* = 0.04) ^37^.

Several new antibiotics with activity against MDR *P. aeruginosa* have become available recently or are in late stages of development. Ceftolozane/tazobactam is a combination of a novel cephalosporin with a modified side chain and a β-lactamase inhibitor. The potent anti-pseudomonal activity of ceftolozane/tazobactam is attributed to its ability to evade the resistance mechanisms of *P*. *aeruginosa*, including efflux pumps, reduced uptake through porins, and modification of penicillin-binding proteins (PBPs) ^38^. A higher dose (3 g intravenously [IV] every 8 hours) has been recommended for treating pneumonia compared with the currently approved dose (1.5 g IV every 8 hours) based on pharmacokinetic estimates for achieving a more than 90% probability of target attainment for the 1-log kill target against pathogens with a minimum inhibitory concentration (MIC) of not more than 8 mg/L in epithelial lining fluid ^39^. Of concern is a recent retrospective study from a single center in Germany that reported that 55% of *P. aeruginosa* isolates were resistant to ceftolozane/tazobactam, which the authors suggested may be due to carriage of the VIM-2 carbapenemase ^40^. Notably, ceftolozane/tazobactam lacks activity against Ambler class B (metallo-)carbapenemases, such as VIM and NDM.

Ceftazidime/avibactam, a combination of a third-generation cephalosporin and a novel synthetic non-β-lactam, β-lactamase inhibitor, is also ineffective against metallo-β-lactamases. In pooled data from five randomized controlled trials including one on HAP/VAP (REPROVE), 95 cases of MDR *P. aeruginosa* were identified ^41^. The favorable microbiological response rates at test of cure for MDR *P. aeruginosa* were 57.1% for ceftazidime/avibactam and 53.8% for comparators, primarily carbapenems ^41^. Thus, ceftazidime/avibactam likely has a role as a carbapenem-sparing agent for treating MDR *P. aeruginosa* pneumonia. Recent data suggest that ceftazidime/avibactam is a viable option against strains of MDR *P. aeruginosa* that are resistant to ceftolozane/tazobactam ^42^.

Cefiderocol is a novel siderophore cephalosporin that inhibits cell wall synthesis through the formation of an iron ion complex that penetrates bacteria via a ferric iron transporter system ^43^. Cefiderocol has demonstrated potent activity against β-lactamase-producing *P. aeruginosa*, including those expressing ESBLs, Ambler class A β-lactamases, and metallo-β-lactamases ^44, 45^. Currently, there is a randomized clinical trial under way for the treatment of HAP comparing the combination of cefiderocol and linezolid with linezolid and meropenem (ClinicalTrials.gov Identifier: NCT03032380) ^46^. The novel β-lactamase inhibitor relebactam inhibits Ambler class A and C β-lactamases and is in development in combination with imipenem. According to data from a global surveillance program that included Africa, Asia, Europe, Latin America, the Middle East, US/Canada, and the South Pacific, imipenem/relebactam inhibited 90.8% of all *P. aeruginosa* isolates and 70.7% of MDR *P. aeruginosa* isolates (n = 3,708) ^47^. A study on patients with HAP/VAP is ongoing. Finally, though still in early development, murepavadin is the first member of a novel class of outer membrane protein-targeting antibiotics specifically designed to target *P. aeruginosa* ^48^. In a study that included 785 isolates of extremely drug-resistant *P. aeruginosa*, Sader *et al*. reported that murepavadin showed potent activity against isolates that were non-susceptible to colistin, ceftolozane/tazobactam, or tobramycin or a combination of these ^49^. These promising findings raise hopes for the further development of murepavadin.

## *Acinetobacter baumannii* 

Most cases of *Acinetobacter baumannii* pneumonia occur in hospitalized patients, although it is occasionally seen in CAP ^50^. Therefore, the recent report that, after increasing for many years, the rate of AMR in *A. baumannii* hospital-acquired infections may be leveling off is grounds for cautious optimism ^51^. AMR in *A. baumannii* is the primary reason that clinicians prescribe ineffective empirical antibiotic therapy, often leading to poor outcomes ^52^. For example, VAP due to MDR *A. baumannii* results in significantly lower rates of successful ventilator weaning compared with susceptible strains ^53^. A retrospective cohort study that included 175 hospitals found that having pneumonia or sepsis from MDR *A. baumannii* was significantly associated with receiving inappropriate antibiotic therapy and higher hospital mortality ^54^. Thus, it is crucial that risk factors for MDR *A. baumannii* be recognized early so that appropriate empiric therapy can be rapidly initiated.

When pneumonia due to MDR *A. baumannii* is suspected (that is, during an *A. baumannii* outbreak, in an endemic setting, or in a previously colonized patient), combination therapy including a polymyxin should be empirically prescribed until susceptibilities are known ^55^. If clinical suspicion for resistance is low, then a carbapenem (except ertapenem, which lacks activity against *A. baumannii*) should be first-line therapy. Many combination therapies for MDR *A. baumannii* have been investigated and were recently discussed by Vazquez Guillamet and Kollef ^6^. Polymyxins remain the backbone of combination regimens. A retrospective cohort study that included patients with pneumonia caused by strains of *A. baumannii* susceptible only to colistin and tigecycline compared three combination regimens: colistin and high-dose sulbactam (n = 93), colistin and tigecycline (n = 43), and colistin and high-dose prolonged infusion of a carbapenem (n = 30) ^56^. The 28-day survival rate and mean length of hospital stay were not statistically different between regimens, whereas an elevated Acute Physiologic Assessment and Chronic Health Evaluation (APACHE) score, delay in receipt of an active regimen, underlying malignancy, and chronic kidney disease were all significantly associated with increased mortality ^56^. Using a loading dose of IV colistin for MDR *A. baumannii* VAP appears to have no significant effect on clinical cure rates or bacteriologic clearance but does increase the risk for nephrotoxicity ^57^. The addition of inhaled colistin to systemic therapies has generally showed favorable results, including better microbiological response. A prospective observational study that compared an IV β-lactam plus IV aminoglycoside, monotherapy with inhaled colistin, and aerosolized colistin plus IV aminoglycoside found no difference in cure rates ^58^. Another study in which IV colistin was compared with IV colistin plus inhaled colistin and inhaled colistin alone also found no differences in mortality or clinical cure, and microbiological cure was better in the aerosolized group ^59^. Once susceptibility results of *A*. *baumannii* isolates become available, combination therapy may be de-escalated to monotherapy. However, tigecycline alone should be avoided, as resistance in *A. baumannii* can develop rapidly ^60^.

Although there have been several recent approvals of antibiotics with activity against Gram-negative pathogens (for example, delafloxacin, ceftazidime-avibactam, ceftolozane-tazobactam, and meropenem-vaborbactam), the pipeline is limited for agents effective against MDR *A. baumannii* ( Table 1). Cefiderocol has been shown to have potent *in vitro* activity against *A. baumannii* and exhibits high stability against carbapenemase hydrolysis ^61^. ** A 52-country collection of clinical isolates obtained between 2014 and 2016 found that 330/368 (89%) of MDR *A*. *baumannii* strains had cefiderocol MICs of not more than 4 μg/mL ^62^. Another study evaluated 107 carbapenem-resistant *A*. *baumannii* isolates from 18 Greek hospitals and determined that the MIC _90_ of cefiderocol was 0.5 mg/L, which was more active than either tigecycline or colistin ^63^. Two phase III clinical trials for cefiderocol—APEKS-NP and CREDIBLE-CR—that include VAP and HAP due to GNB are under way ^64^.

**Table 1.** New antibiotics for multidrug-resistant Gram-negative bacteria.

Drug	Class	Development stage	Activity	FDA indication
Aztreonam/avibactam	Monobactam/β-lactamase inhibitor	Phase II	ESBL, KPC, class C β-lactamase, MBL	Not applicable
Cefiderocol	Siderophore cephalosporin	Phase III	ESBL, CRE (class A, B, and D enzymes), carbapenem-resistant *Pseudomonas* *aeruginosa*, *Stenotrophomonas* *maltophilia*, and *Acinetobacter baumannii*	Not applicable
Ceftazidime/avibactam	Cephalosporin/β- lactamase inhibitor	FDA-approved	ESBL, KPC, AmpC, some class D serine β-lactamases	HABP/VABP, cIAI, cUTI
Ceftolozane/tazobactam	Cephalosporin/β- lactamase inhibitor	FDA-approved	ESBL, MDR *P. aeruginosa*	cUTI, cIAI
Delafloxacin	Fluoroquinolone	FDA-approved	*Klebsiella pneumoniae*, including AmpC and class A ESBL-producers, ciprofloxacin-resistant *Escherichia coli* and *A. baumannii*	ABSSSI
Eravacycline	Fluorocycline tetracycline	FDA-approved	ESBL, CRE, MDR *A. baumannii*	cIAI
Imipenem+cilastatin/ relebactam	Carbapenem/β-lactamase inhibitor	Phase III	KPC, MDR *P. aeruginosa*	Not applicable
Meropenem/vaborbactam	Carbapenem/boronic acid inhibitor	FDA-approved	CRE (class A and C enzymes)	cUTI
Murepavadin	Cyclic peptide that targets outer membrane	Phase III	MDR *P. aeruginosa*	Not applicable
Omadacycline	Aminomethylcycline	FDA-approved	ESBL, *A. baumannii*	ABSSSI, CABP
Plazomicin	Aminoglycoside	FDA-approved	ESBL, CRE excluding NDM producers, *A. baumannii*, *P. aeruginosa*	cUTI

ABSSSI, acute bacterial skin and skin structure infection; CABP, community-acquired bacterial pneumonia; cIAI, complicated intra-abdominal infection; CRE, carbapenem-resistant Enterobacteriaceae; cUTI, complicated urinary tract infection; ESBL, extended-spectrum β-lactamase; FDA, US Food and Drug Administration; HABP, hospital-acquired bacterial pneumonia; KPC, *Klebsiella pneumoniae* carbapenemase; MBL, metallo-β-lactamase; MDR, multidrug-resistant; NDM, New Delhi metallo-β-lactamase; VABP, ventilator-acquired bacterial pneumonia.

Eravacycline was recently approved for the treatment of intra-abdominal infections and complicated urinary tract infections (cUTIs). One study showed that against carbapenem- and tigecycline-resistant *Acinetobacter* isolates, eravacycline MICs were about twofold lower versus comparator agents ^65^. However, clinical experience with eravacycline for *A. baumannii* pneumonia is limited and its role is unclear, especially given evidence of increased adverse events and mortality with tigecycline when prescribed for pneumonia ^66^.

Plazomicin is a novel aminoglycoside derived from sisomicin that was modified to resist aminoglycoside-modifying enzymes and is currently approved for use in cUTIs. Significantly improved activity has been observed in OXA-producing *A*. *baumannii* compared with other aminoglycosides, and plazomicin MICs are 16 to 32 times lower ^67^. A recent clinical trial found favorable results with plazomicin in CRE VAP as well as a favorable safety profile and a low incidence of drug-related adverse events, including serum creatinine elevations ^68^. Thus, plazomicin appears promising as part of combination regimens and data on its effectiveness in HAP/VAP due to MDR *A. baumannii* are eagerly awaited.

Zidebactam and WCK 5153 are two novel β-lactam antibiotics under development that display high affinity for PBP2 of GNB and overcome multiple β-lactam resistance mechanisms. When combined with cefepime or sulbactam, zidebactam and WCK 5153 demonstrated enhanced killing and full bacterial eradication after 24 hours against strains of MDR *A. baumannii* ^69^. Arylomycins are a new class of lipopeptide antibiotics whose lead compound, G0775, was demonstrated to have potent activity against 16 strains of MDR *A. baumannii* ^70^. The antibiotic adjuvant SPR741 sensitized *A. baumannii* to a panel of antibiotics and permitted strong potentiation of rifampin against multiple MDR *A. baumannii* isolates ^71^. Several non-antibiotic therapies against *A. baumannii* are in various stages of development (for example, bacteriophage, vaccines, and monoclonal antibodies) and have recently been reviewed ^72^. Finally, it may be possible in the future to use CRISPR-Cas systems to target plasmids that spread AMR in GNB ^73^.

## Conclusions

Pneumonia due to MDR-GNB represents a serious threat to hospitalized patients. Clinicians must be knowledgeable about local resistance patterns and a patient’s risk factors for MDR-GNB to ensure appropriate empiric antimicrobial therapy. Fortunately, several new drugs that target MDR-GNB have been approved or are in late stages of development. Further pragmatic studies are needed to elucidate their place in therapy and their impact on real-world outcomes such as length of stay and mortality, especially for ICU patients with HAP/VAP.
